# Preoperative CT versus intraoperative hybrid DynaCT imaging for localization of small pulmonary nodules: a randomized controlled trial

**DOI:** 10.1186/s13063-019-3532-z

**Published:** 2019-07-04

**Authors:** Yin-Kai Chao, Kuang-Tse Pan, Chih-Tsung Wen, Hsin-Yueh Fang, Ming-Ju Hsieh

**Affiliations:** 1 Division of Thoracic Surgery Chang Gung Memorial Hospital-Linko, College of Medicine Chang Gung University, Taoyuan, Taiwan; 2 Department of Medical Imaging and Intervention College of Medicine, Chang Gung University, Taoyuan, Taiwan

**Keywords:** Hybrid operating room, Slitary pulmonary nodules, Localization, ARTIS zeego

## Abstract

**Background:**

Localization of small and/or deep pulmonary nodules before thoracoscopic exploration is paramount to minimize the likelihood of unplanned conversion to thoracotomy. As far as the percutaneous approach is concerned, the most common workflow consists of preoperative computed tomography (POCT) imaging-guided tumor marking (performed in an interventional CT suite) followed by their removal in an operating room (OR). However, the advent of hybrid ORs has allowed intraoperative computed tomography (IOCT)-guided lesion localization. This single center, open-label, randomized, controlled clinical trial aims to compare the efficacy and safety of IOCT versus POCT.

**Methods/design:**

The study sample will consist of patients presenting with small and/or deep pulmonary nodules who will be randomly allocated to either POCT or IOCT. The time required to complete lesion localization will be the primary efficacy outcome. The following parameters will serve as secondary endpoints: rate of successful targeting during localization and in the operating field, time at risk, operating time, length of time under anesthesia, global OR utilization time, complication (pneumothorax and hemorrhage) rates, and radiation exposure.

**Discussion:**

Owing to the increased availability of HORs, our data will be crucial to clarify the feasibility and safety of IOCT versus the traditional POCT approach.

**Trial registration:**

ClinicalTrials.gov, NCT03395964. Registered on October 8, 2018.

**Electronic supplementary material:**

The online version of this article (10.1186/s13063-019-3532-z) contains supplementary material, which is available to authorized users.

## Background

With the implementation of lung cancer screening based on low-dose computed tomography (CT), the number of patients diagnosed with small and/or deeply located pulmonary nodules has markedly increased. Lesions detected during screening should be carefully evaluated and eventually removed (when their malignant nature is highly suspected) [1]. However, their excision through video-assisted thoracoscopic surgery (VATS) may be challenging, particularly when such nodules are small (< 10–15 mm in size) and/or deeply located (> 5–10 mm from the pleural surface) in the lung parenchyma [2, 3]. Efficient and safe tumor marking before embarking on VATS is paramount to avoid an unplanned conversion to thoracotomy [4]. To this aim, several approaches have been proposed, including percutaneous CT-guided [5–8], bronchoscopy-guided (grounded in segmental anatomy and virtual imaging) [9, 10], and electromagnetic navigation bronchoscopy (ENB)-guided [11–13] methods. The results of a randomized study have previously shown that preoperative lesion localization is superior to no localization in terms of increased number of successful VATS wedge resections, reduced surgical time, and less frequent use of staples [3]. Moreover, total costs did not increase appreciably.

As far as the percutaneous approach is concerned, the most common workflow consists of preoperative computed tomography (POCT) imaging-guided tumor marking (performed in an interventional CT suite) followed by their removal in an operating room (OR) [5, 8]. However, this two-step methodology requires careful planning in order to avoid complications (e.g., pneumothorax, hemothorax, wire dislodgement, dye diffusion), the incidence of which increases in parallel with the time elapsed from localization to surgery.

The recent advent of hybrid ORs is triggering a paradigm shift in the treatment of pulmonary nodules, paving the way to intraoperative CT (IOCT)-guided VATS. Although numerous studies have already shown that IOCT is clinically feasible [14–16], the question as to whether this approach is superior to traditional POCT remains open [17]. To address this issue, well-designed prospective randomized studies are eagerly awaited [18]. Here, we describe the protocol of a single-center prospective study that aims to provide a direct head-to-head comparison of IOCT versus POCT for localizing pulmonary nodules. The two techniques will be investigated in relation to efficacy, accuracy, complications, and radiation exposure.

## Methods/design

### Study design

**Fig. 1:**
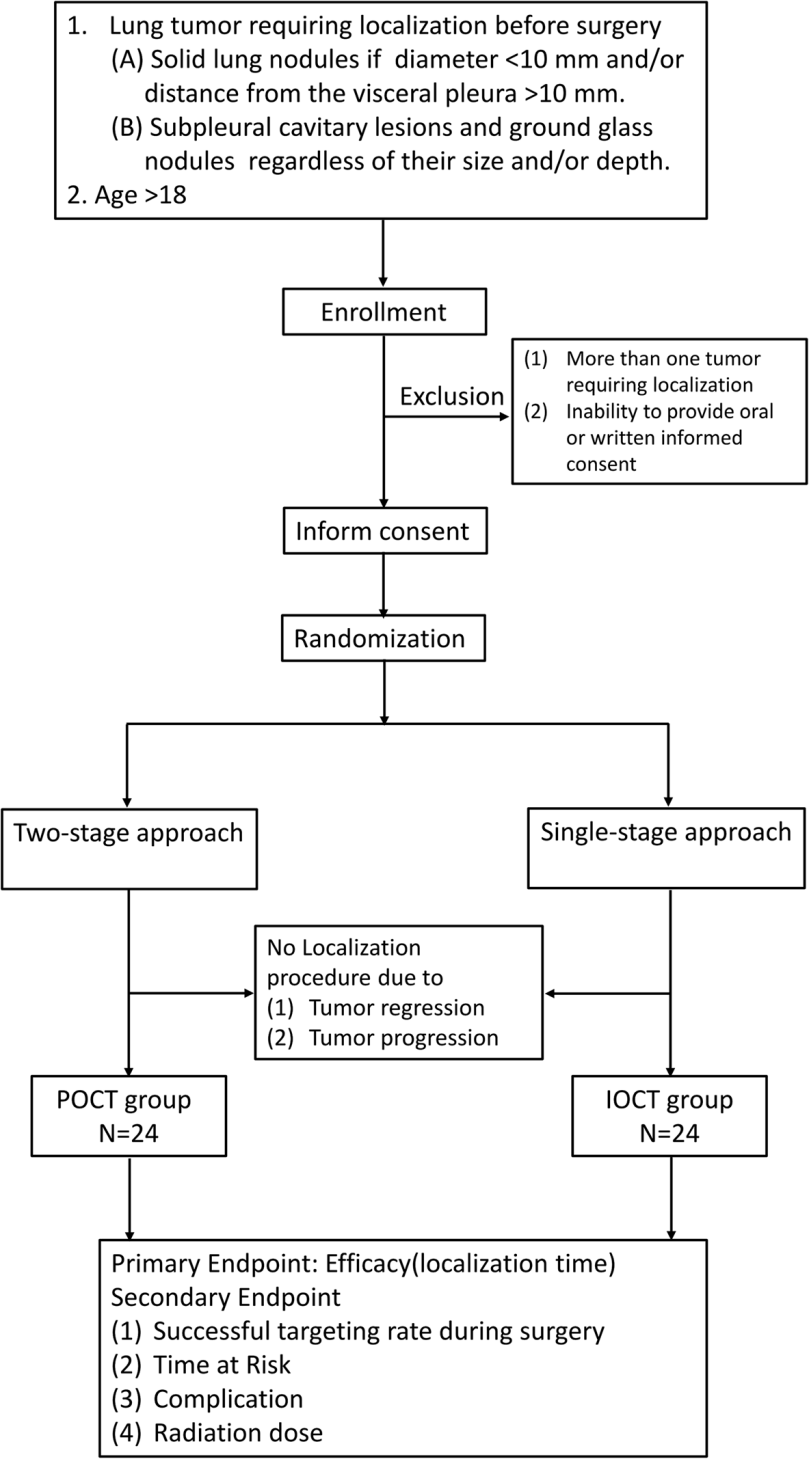
Flowchart of the study

Figure 1 depicts the flow of this investigator-initiated, investigator-driven study, which was designed as a single-center, open-label, randomized controlled trial. Patients diagnosed with pulmonary nodules and scheduled to undergo tumor localization before VATS will be randomized (1:1 ratio) to either IOCT or POCT.

### Study patients

Subjects aged at least 18 years will be considered eligible for the study if they have a lung tumor requiring localization before VATS. Localization will be deemed necessary when one of the following criteria is met: 1) presence of solid pulmonary nodules of less than 10 mm in size and/or with a distance from the visceral pleura to the edge of nodule of at least 10 mm; or 2) evidence of subpleural cavitary lesions/ground glass nodules (GGNs), independent of their size and/or depth.

Exclusion criteria will be as follows: 1) presence of more than one nodule requiring localization; 2) inability to provide written informed consent; and 3) unwillingness to adhere to the proposed follow-up protocol.

### Screening for inclusion

Potentially eligible patients will be approached about potential inclusion either during the prehospitalization visit or while being hospitalized (before scheduled surgery). Complete information about the study objectives will be provided to all candidates.

### Randomization

Patients will be randomized to either IOCT or POCT (1:1 ratio) using a computerized randomization tool. We will implement a permuted-block randomization scheme with varying block sizes, while maintaining both allocation and block sizes concealed to the study investigators. Owing to the obvious procedural differences between IOCT and POCT, blinding of surgeons and patients cannot be achieved. In order to ensure an objective evaluation of the endpoints, all of the study outcomes will be investigated by an independent assessor (blinded to the allocation of patients to either POCT or IOCT) through a careful review of clinical records. After randomization, patients will be excluded when tumor regression or progression will be evident on prelocalization images (ultimately abrogating the need of localization). Patients will be allowed to exit from the study at any time.

### POCT-guided localization

**Fig. 2:**
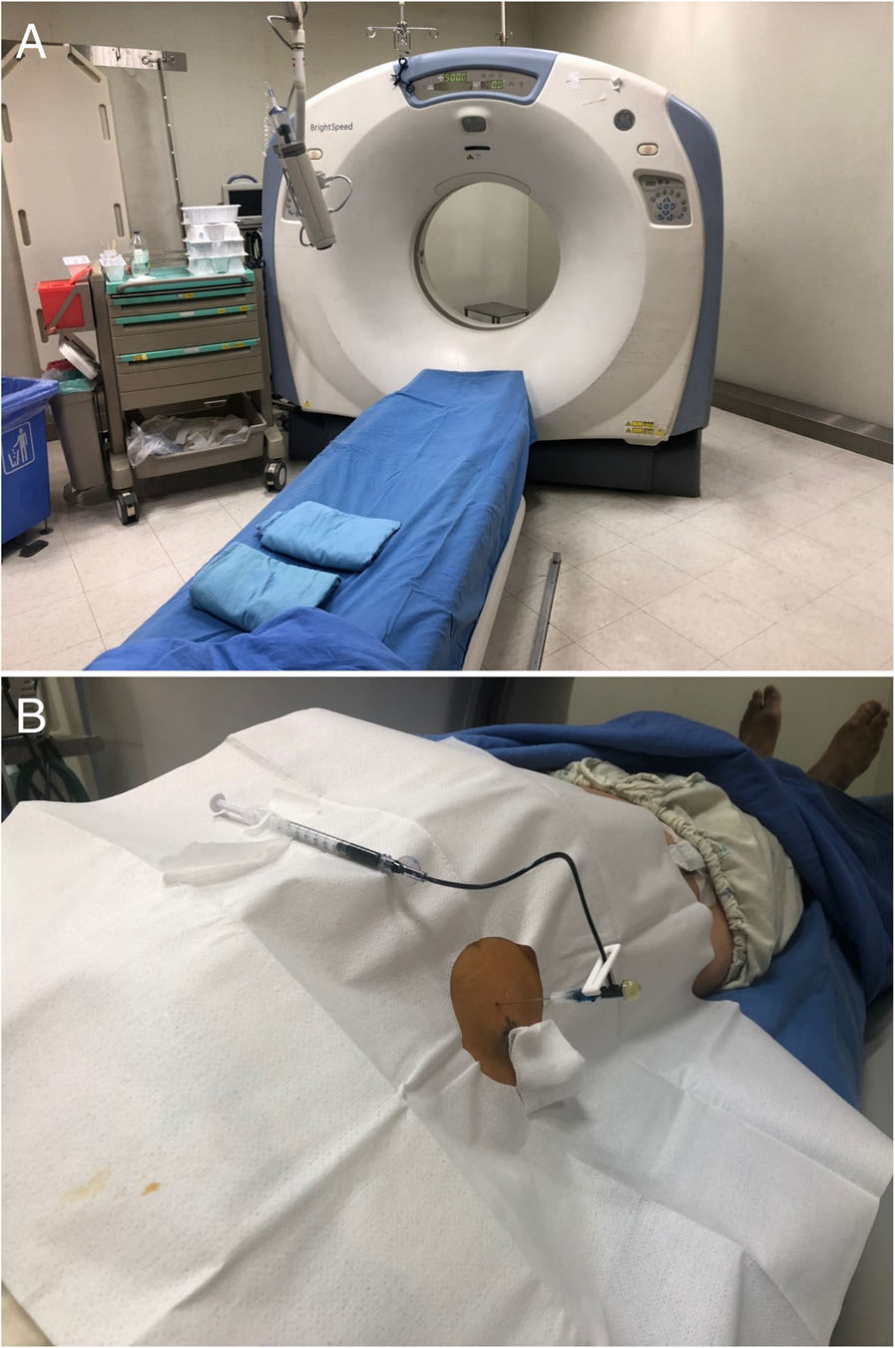
**a** Interventional radiology suite equipped with a 16-slice CT scanner (GE BrightSpeed; GE Healthcare, Milwaukee, WI, USA). **b** PBV dye injection following needle localization

All POCT-guided localization will be performed on a CT scanner (GE BrightSpeed; GE Healthcare, Milwaukee, WI, USA; Fig. 2a) by a single team of interventional radiologists according to a previously described workflow [8]. Before implementing the procedure, case images will be reviewed to determine the most suitable needle trajectory. Sterile wraps will be positioned around the patient’s chest, followed by injection of 1% lidocaine at the site of needle insertion in the chest. A scalpel will be subsequently used to create a small skin incision followed by the insertion through the chest wall of a 10.7-cm-long, 20-gauge cannula needle containing a double-thorn hookwire (length 20 cm; DuaLok®, Bard Peripheral Vascular Inc., Tempe, AZ, USA). Under intermittent CT guidance, the needle will be positioned at the edge of the nodule of interest. As soon as the needle tip is close to or reaches the lesion, the hookwire will be advanced through the cannula. PBV dye (0.5 mL, patent blue V 2.5%; Guerbet, Aulnay-sous-Bois, France) injected through a 22-gauge, 8.9-long spinal needle will be used to localize superficial lesions (Fig. 2b). The proper reciprocal positioning of the lesion and hookwire will be investigated through an immediate follow-up CT scan. Upon completion of localization, patients will be moved to a general ward before undergoing the scheduled resection.

### IOCT-guided localization

**Fig. 3:**
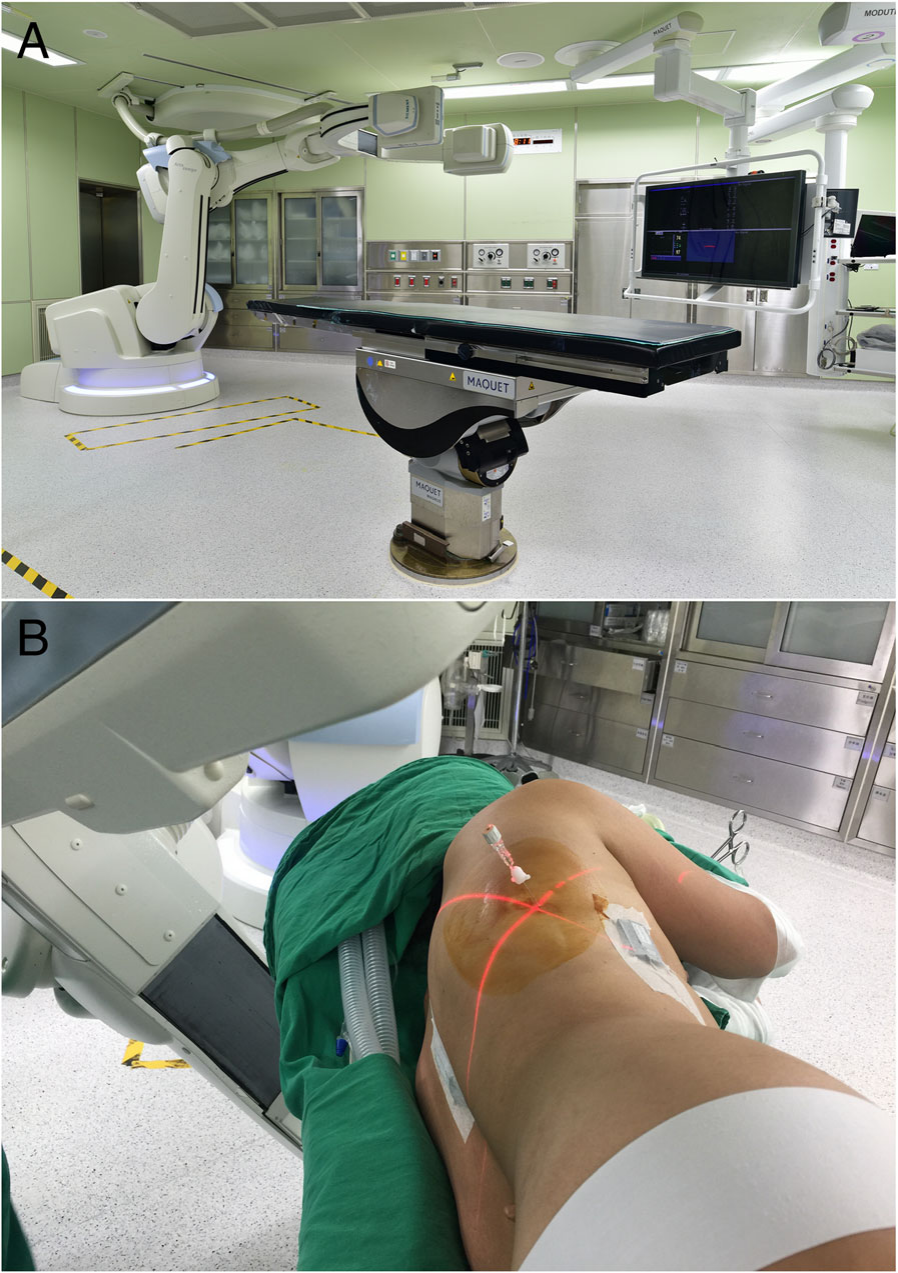
**a** Hybrid operating room equipped with a cone-beam CT apparatus (ARTIS zeego; Siemens Healthcare GmbH, Erlangen, Germany) and a Magnus surgical table (Maquet Medical Systems, Wayne, NJ, USA). **b** The needle entry point and angulation were visualized by projecting a laser-targeting cross onto the patient’s surface

Patients in the IOCT group will undergo lesion localization in a hybrid OR equipped with C-arm cone-beam computed tomography (CBCT; ARTIS zeego; Siemens Healthcare GmbH, Erlangen, Germany) and a Magnus surgical table (Maquet Medical Systems, Wayne, NJ, USA) (Fig. 3a). The nodules will be localized and subsequently removed in a unique section by a single team of thoracic surgeons according to a previously described workflow [19]. After induction of general anesthesia, patients will be positioned in the lateral decubitus. A 6-s protocol (6 s DynaCT Body) will be used to acquire an initial scan for surgical planning (with the patient under end-inspiratory breathhold). We will model the needle entry path in the isotropic data set under the syngo Needle Guidance provided with the syngo X-Workplace (Siemens Healthcare GmbH). The needle trajectory will be initially identified by marking the entry and target points. A laser-target cross will be projected onto the patient’s surface to visualize the needle entry point and angulation. An 18-gauge marker needle will be deployed into the patient’s thorax during end-inspiratory breathhold under three-dimensional laser guidance and guided fluoroscopy (Fig. 3b). CBCT will be used to confirm an appropriate needle positioning, and the lesion will be subsequently localized using either a hookwire (DuaLok®; Bard Peripheral Vascular Inc.) or a microcoil (Cook Medical, Bloomington, IN, USA). Superficial lesions will be identified through the injection of either PBV (0.3–0.5 mL, patent blue V 2.5%; Guerbet, Villepinte, France) or near-infrared dye as previously described [20].The correct lesion localization will be confirmed through a post-procedural CBCT scan.

### Surgical treatment

Patients in both arms will undergo VATS wedge resection, with the resected specimen being submitted to frozen section examination. Cases with a confirmed diagnosis of primary lung cancer will undergo lobectomy. Patients unable to tolerate lobectomy because of an inadequate pulmonary function or with peripheral lung cancer of limited size (< 2 cm) and adequate resection margins (either > 2 cm or bigger than tumor size) will be treated with a sublobar resection (wedge resection or segmentectomy).

### Data collection and management

Each participant will be unequivocally identified through a personal code (accessible to the principal investigator and the study coordinators only) assigned at inclusion. Digital case record forms (CRF) compliant with good clinical practice standards will be used for data collection and managed by the study coordinators and/or research nurses. Paper records will be stored in secured cabinets located at the data coordinating centers, with access being granted to the principal investigator and other researchers (nurses and physicians). Request for consultation of raw data (upon completion of the study) should be directed to the principal investigation. In order to ensure that the primary and secondary study outcomes will be accurately reported, all CRFs will be thoroughly cross-checked with the original sources. Clinical data will be stored in an anonymized fashion in keeping with local privacy laws.

### Primary outcome measure

The time required for lesion localization will be the primary outcome measure. In the POCT group, it will be defined as the time elapsed from the beginning of preprocedural CT imaging to the end of postprocedural CT scan. In the IOCT group, it will be calculated from the docking of the C-arm to the end of the procedure (i.e., retraction of the C-arm from the table to the park position).

### Secondary endpoints

The following secondary endpoints will be examined: 1) successful targeting rates during localization (defined as the number of successful targeting procedures divided by the number of all localization procedures); 2) successful targeting rates in the operating field (defined as the number of successful targeting procedures minus the number of wire dislodgements or dye fading/spillage occurring in the operation field divided by the number of all localization procedures); 3) time at risk (defined as the time elapsed between the completion of localization and skin incision); 4) other time parameters (including operating time, length of time under anesthesia, global operation room utilization time, and length of hospital stay); 5) rate of conversion to thoracotomy; and 6) complication rates. The occurrence of complications (including pneumothorax and lung hemorrhage) will be recorded after the initial follow-up CT scan following localization. According to the 2010 British Thoracic Society guidelines, large or small pneumothorax will be defined by a distance between the lung margin and chest wall greater or less than 2 cm, respectively [21]. As far as radiation doses are concerned, we will quantify the radiation dose delivered to patients by determining the effective dose (ED). During POCT procedures, the radiation dose delivered by MDCT will be determined using the dose length product (DLP) and expressed as mGy/cm. The radiation dose will be converted to the ED using a suitable conversion factor (0.014, mSvGy^− 1^ cm^− 1^) [22]. During IOCT procedures, the radiation doses delivered by both CBCT and fluoroscopy will be determined using the dose area product (DAP) and expressed as mGy/cm^2^. Two appropriate conversion factors (0.146 and 0.12 mSvGy^− 1^ cm^− 2^) will be used to calculate the ED for CBCT and fluoroscopy, respectively [23, 24]. Four sets of thermoluminescent dosimeters (TLDs; UD-802A; Panasonic, Osaka, Japan) will be also placed around the patient’s chest wall (in proximity to the lesion of interest). The radiation dose absorbed by each TLD will be measured using a TLD reader (UD-716AGL TLD reader; Panasonic, Tokyo, Japan) and mean values will be used for analysis.

### Follow-up schedule

**Fig. 4:**
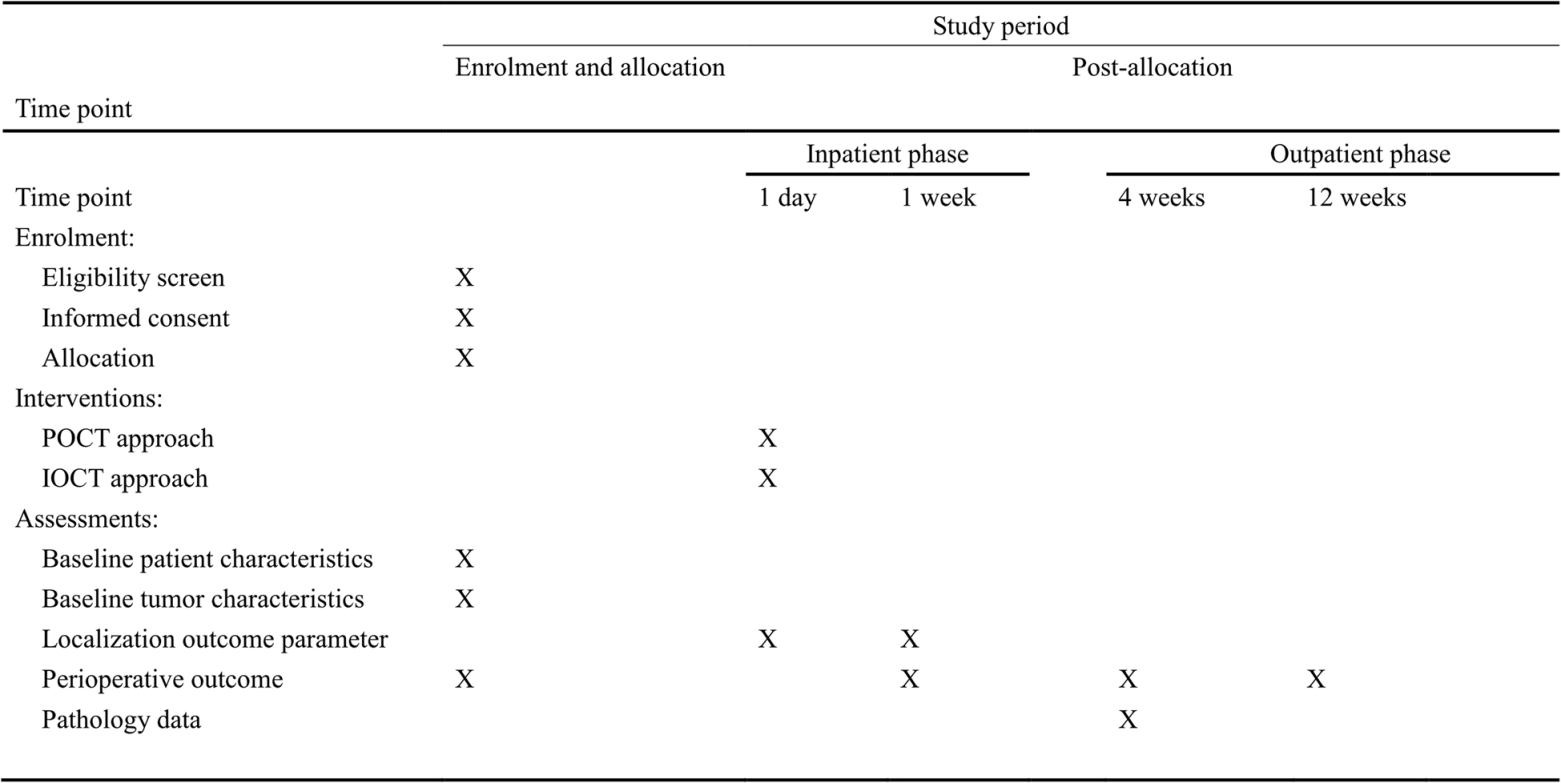
Schedule of patient enrolment, interventions, and assessments; Standard Protocol Items: Recommendations for Interventional Trials (SPIRIT)

The start of the study will be set at randomization. Follow-up will be performed until 3 months after surgery according to a predetermined schedule (Fig. 4). Within one week of the operation, we will assess the primary study endpoint. The following variables will be collected: postoperative complications, readmission rates, and deaths occurring within 30 and 90 postoperative days. Postoperative visits will be scheduled at 3–4 weeks after surgery and at 3 postoperative months.

### Sample size calculation

The sample size was established according to a retrospective study previously designed by our group [25]. Our original assumption was that the time required for tumor localization would be the same in the IOCT and POCT groups. Based on a two-sample *t*-test under an equality hypothesis, at least 24 patients per treatment arm will be required under the following conditions: alpha error, 0.05; power, 80%; and a balanced trial design. Under the assumption of a 10% total dropout rate, we are planning to enroll at least 27 patients in each arm.

### Timeline

The clinical trial will last two years, a time span that includes prearrangement and statistical analysis. Recruitment began on October 8, 2018, with a planned 2-year duration. Data analysis is scheduled to start upon discharge of the last randomized patient.

### Data analysis

Both intention-to-treat (i.e., in all of the randomized patients) and per-protocol (i.e., only in patients who will have their pulmonary lesion localized according to the method assigned on randomization and with complete follow-up data) analyses will be conducted. Categorical variables will be expressed as frequencies and compared with the chi-squared test or the Fisher’s exact test, as appropriate. Continuous data will be summarized as means ± standard deviations (for Gaussian variables) or medians and interquartile ranges (for skewed parameters). The Mann-Whitney *U* test and the Student’s *t*-test will be used to compare normally distributed and skewed continuous variables, respectively. Two software packages—SAS (version 9.3; SAS Institute Inc., Cary, NC, USA) and SPSS (version 20.0; SPSS Inc., Chicago, IL, USA)—will be used for statistical calculations. A *P* value < 0.05 (two-tailed) will be considered statistically significant.

## Discussion

Currently, two major techniques can be implemented for performing CT-guided VATS removal of pulmonary nodules. The first is a two-stage approach based on preoperative lesion localization in a CT suite followed by its excision in an operating room, whereas the second consists in single-stage localization and removal in a hybrid OR.

It is a common assumption that IOCT-guided VATS performed in a hybrid OR may ultimately reduce the time at risk between localization and the subsequent excision when compared to POCT—ultimately resulting in a more patient-centered approach. The current randomized clinical trial will be the first to test the hypothesis that, besides reducing the time at risk, IOCT-guided VATS could also be as effective as the conventional two-stage POCT-guided approach for localizing pulmonary nodules. In particular, we will focus on the time required for localization when each approach will be used (a variable which will serve as the primary study endpoint). In addition, we will compare the successful targeting rates during surgery. In terms of safety, a point that will merit consideration is the radiation exposure delivered to patients. Because CBCT and MDCT differ significantly in terms of radiation dynamics, direct use of scanner-estimated doses will be suboptimal for comparison purposes. In order to circumvent this issue, patients in the IOCT arm will be requested to apply TLDs. This approach will allow obtaining direct measures of individual surface radiation exposure. Finally, we are aware that the utilization of the IOCT-guided approach in a hybrid OR may potentially increase the procedural costs (owing to a longer time under anesthesia and a higher global OR utilization time). The question as to whether IOCT will be cost-effective compared with POCT will be assessed separately.

### Trial status

The trial commenced on October 8, 2018 and the recruitment period is projected to last 2 years. Data analysis will be started upon discharge of the last randomized patient.

## Additional file

Additional file 1: SPIRIT 2013 checklist: Recommended items to address in a clinical trial protocol and related documents*. (DOC 121 kb)

## Data Availability

Raw data will be available from the corresponding author upon reasonable request. Transfer of clinical data will require approval from the Institutional Review Board.

## Abbreviations

CBCT: Cone-beam computed tomography
CRF: Case record form
CT: Computed tomography
ENB: Electromagnetic navigation bronchoscopy
GCP: Good clinical practice
GGNs: Ground glass nodules
HORs: Hybrid operating rooms
IOCT: Intraoperative computed tomography
OR: Operating room
POCT: Preoperative CT
SPIRIT: Standard Protocol Items: Recommendations for Interventional Trials
TLDs: Thermoluminescent dosimeters
VATS: Video-assisted thoracoscopic surgery

## References

[1] Team NLSTR (2011). Reduced lung-cancer mortality with low-dose computed tomographic screening. N Engl J Med.

[2] Suzuki K, Nagai K, Yoshida J (1999). Video-assisted thoracoscopic surgery for small indeterminate pulmonary nodules. Chest.

[3] Finley RJ, Mayo JR, Grant K (2015). Preoperative computed tomography–guided microcoil localization of small peripheral pulmonary nodules: a prospective randomized controlled trial. J Thorac Cardiovasc Surg.

[4] Detterbeck FC, Homer RJ (2011). Approach to the ground-glass nodule. Clin Chest Med.

[5] Lin M-W, Tseng Y-H, Lee Y-F (2016). Computed tomography-guided patent blue vital dye localization of pulmonary nodules in uniportal thoracoscopy. J Thorac Cardiovasc Surg.

[6] Ichinose J, Kohno T, Fujimori S, Harano T, Suzuki S (2013). Efficacy and complications of computed tomography-guided hook wire localization. Ann Thorac Surg.

[7] Thistlethwaite PA, Gower JR, Hernandez M (2018). Needle localization of small pulmonary nodules: Lessons learned. J Thorac Cardiovasc Surg.

[8] Chen Y-R, Yeow K-M, Lee J-Y (2007). CT-guided hook wire localization of subpleural lung lesions for video-assisted thoracoscopic surgery (VATS). J Formos Med Assoc.

[9] Sato M, Omasa M, Chen F (2014). Use of virtual assisted lung mapping (VAL-MAP), a bronchoscopic multispot dye-marking technique using virtual images, for precise navigation of thoracoscopic sublobar lung resection. J Thorac Cardiovasc Surg.

[10] Sato M, Kuwata T, Yamanashi K (2017). Safety and reproducibility of virtual-assisted lung mapping: a multicentre study in Japan. Eur J Cardiothorac Surg.

[11] Abbas A, Kadakia S, Ambur V (2017). Intraoperative electromagnetic navigational bronchoscopic localization of small, deep, or subsolid pulmonary nodules. J Thorac Cardiovasc Surg.

[12] Anayama T, Qiu J, Chan H (2015). Localization of pulmonary nodules using navigation bronchoscope and a near-infrared fluorescence thoracoscope. Ann Thorac Surg.

[13] Marino KA, Sullivan JL, Weksler B (2016). Electromagnetic navigation bronchoscopy for identifying lung nodules for thoracoscopic resection. Ann Thorac Surg.

[14] Kostrzewa M, Kara K, Rathmann N (2017). Computed tomography-assisted thoracoscopic surgery: A novel, innovative approach in patients with deep intrapulmonary lesions of unknown malignant status. Investig Radiol.

[15] Yang S-M, Ko W-C, Lin M-W (2016). Image-guided thoracoscopic surgery with dye localization in a hybrid operating room. J Thorac Dis.

[16] Hsieh M-J, Fang H-Y, Lin C-C (2017). Single-stage localization and removal of small lung nodules through image-guided video-assisted thoracoscopic surgery. Eur J Cardiothorac Surg.

[17] Cameron R (2018). Interventional radiology suite or hybrid operating room: Which is the best for lung nodule localization?. J Thorac Cardiovasc Surg.

[18] Weksler B (2017). Time for randomization. J Thorac Cardiovasc Surg.

[19] Hsieh C-P, Hsieh M-J, Fang H-Y, Chao Y-K (2017). Imaging-guided thoracoscopic resection of a ground-glass opacity lesion in a hybrid operating room equipped with a robotic C-arm CT system. J Thorac Dis.

[20] Wen CT, Liu YY, Fang HY (2018). Image-guided video-assisted thoracoscopic small lung tumor resection using near-infrared marking. Surg Endosc.

[21] MacDuff A, Arnold A, Harvey J (2010). Management of spontaneous pneumothorax: British Thoracic Society pleural disease guideline 2010. Thorax.

[22] American Association of Physicists in Medicine. AAPM report 96. The Measurement, Reporting and Management of Radiation Dose in CT: Report of AAPM Task Group 23 of the Diagnostic Imaging Council CT Committee. College Park, MD American Association of Physicists in Medicine; 2008.

[23] Wielandts J-Y, De Buck S, Ector J (2009). Three-dimensional cardiac rotational angiography: effective radiation dose and image quality implications. Europace.

[24] Schauer DA, Linton OW (2009). NCRP report no. 160. Ionizing radiation exposure of the population of the United States, medical exposure—are we doing less with more, and is there a role for health physicists?. Health Phys.

[25] Chao YK, Pan KT, Wen CT (2018). A comparison of efficacy and safety of preoperative versus intraoperative computed tomography-guided thoracoscopic lung resection. J Thorac Cardiovasc Surg.

